# Evaluation of Patients Presenting With First Febrile Seizure

**DOI:** 10.7759/cureus.16151

**Published:** 2021-07-03

**Authors:** Mahmut Aslan

**Affiliations:** 1 Pediatric Neurology, Mersin City Hospital, Mersin, TUR

**Keywords:** child, high fever, pediatric seizure, risc factors, acute febrile illness

## Abstract

Introduction

Febrile seizure (FS) is the most common cause of convulsion in children. In the present study, we evaluated patients presenting with FS.

Methods

Eighty-two patients aged between 6-60 months who presented to Mersin City Training and Research Hospital with the first febrile seizure between January 2020 and May 2021 were included in the study.

Results

Of the 82 patients included in our study, 42 (51,2%) were male and 40 were female (48,8%). Their average age at presentation of first febrile seizure was 21,05 ± 16,22 months. Fever focus was found in 32 patients (39,1%) with upper respiratory tract infection. Epileptic abnormality was observed in the EEG of six patients (7,3%) and antiepileptic medication was started in three of these patients.

Conclusions

Upper respiratory tract infection, family history of FS, and family history of epilepsy are the main risk factors for the development of FS. Complex FS is a serious risk factor for the development of epilepsy.

## Introduction

Febrile seizure (FS) is the most common cause of convulsion, seen in 2-5% of children [1]. The American Academy of Pediatrics (AAP) defines febrile seizures in healthy patients aged 6-60 months without neuromotor developmental delay. In this patient group, there should be no signs of intracranial infection, intracranial hemorrhage, or metabolic disorder [1]. Febrile seizures are divided into two groups - complex and simple febrile seizures. Simple FS are seizures that last less than 15 minutes and do not recur within 24 hours. Complex FS seizures last longer than 15 minutes and recur more than once in 24 hours [2]. Genetic and environmental factors are two important factors for febrile seizures [3]. Some studies indicate that factors such as hypoxia, premature birth, and birth complications influence the development of FS [4,5]. Febrile seizures are considered benign seizures of childhood. It is known that they do not cause any problems in children later [6].

## Materials and methods

Eighty-two patients aged between 6-60 months who presented to Mersin City Training and Research Hospital with the first febrile seizure between January 2020 and May 2021 were included in the study. The definition of the American Academy of Pediatrics of febrile seizures was followed and used as inclusion/exclusion criteria [1]. Patients with a previous history of epilepsy and seizures, suspected intracranial infection, neuromotor developmental delay, and electrolyte imbalance were not included in the study. We evaluated the demographic and laboratory findings, family history, EEG findings, and prognosis of the patients. Statistical analysis was performed using SPSS version 19 program for windows (IBM Corp., Armonk, USA). All experimental values are presented as mean ± SD or frequency percentages. 

## Results

Of the 82 patients included in our study, 42 (51,2%) were male and 40 were female (48,8%). Their average age at presentation of first febrile seizure was 21,05 ± 16,22 months. First febrile seizure was simple in 38 patients (46,3%) and complex in 44 patients (53,7%). There was consanguinity between the parents of 46 patients (56,1%). Eight patients (9,7%) had a family history of epilepsy, and 16 patients (19,5%) had a family history of febrile seizures. Nine patients (10,9 %) had focal seizures and 73 patients (88,1%) had generalized seizures, and 12 (14,6%) of these patients had status epilepticus. Fever focus was found in 32 patients (39,1%) with upper respiratory tract infection, 14 patients (17,1%) with lower respiratory tract infection, 18 patients (21,9%) with gastroenteritis, six patients (7,3%) with urinary tract infection, and 12 patients (14,6%) with cause unknown (Table 1).

**Table 1:** Demographic and clinical characteristics of the study subjects and their first febrile seizure (N=82) URTI: Upper respiratory tract infection;  LRTI: Lower respiratory tract infection; AGE: Acute gastroenteritis;  UTI: Urinary tract infection

Characteristics	Levels	n	%
Gender	Male	42	51,2
	Female	40	48,2
Consanguinity of the parents	Yes	46	56,1
	No	36	43,9
Family history of epilepsy	Yes	8	9,7
	No	74	90,3
Family history of febrile seizure	Yes	16	19,5
	No	66	80,5
Type of convulsion	Generalized	73	89,1
	Focal	9	10,9
Status epilepticus	Yes	12	14,6
	No	70	85,4
Type of febrile seizure	Simple	38	46,3
	Complex	44	53,7
The source of the febrile illness	URTI	32	39,1
	LRTI	14	17,1
	AGE	18	21,9
	UTI	6	7,3
	Unknown	12	14,6

The mean hemoglobin values of the patients were 11.22 ± 1.1 mg/dl, white cell counts were 14.2 ± 3.2, and C-reactive protein (CRP) values were 1.3 ± 0.62 mg/dl. EEG was taken in 64 of 82 patients (78,1%) within the first three weeks. Epileptic abnormality was observed in the EEG of six patients (7,3%) and antiepileptic medication was started in three of these patients. Four of the six patients with epileptic abnormalities on EEG had complex FS.

## Discussion

Febrile seizures, the most common seizure disorder of childhood, are benign seizures that often do not cause significant long-term side effects. It is more common in early childhood (six months to five years), when the seizure threshold is low, the tendency to infections is more frequent, and the fever response is more intense. In the literature, mostly male predominance has been reported in patients with febrile convulsions. In the series of Okumura et al. covering 203 patients, the male/female ratio was 1.3/1, and in the study of Knudsen, it was 1.4/1 [7,8]. There was also male dominance in our study and the ratio was 1.05. Eighty-two febrile children aged 6-60 months were studied for the risk factors of seizures. The average age was 21,05 ± 16,22 months. Similar studies have also found the same age groups [9]. Some predisposing risk factors for the development of febrile seizures in children have been investigated. A family history of FS, epilepsy, cesarean section, and upper respiratory tract infection (URTI) were recognized as the major risk factors [10,11]. In our study, 24 patients (29.3%) had a family history of epilepsy or FS and 32 of our patients (39.1%) had URTI.

Most of the studies show that while simple febrile seizures are not a risk factor for the development of epilepsy, complex febrile seizures are a risk factor for epilepsy [12-14]. Electroencephalography (EEG) has no place in the diagnosis and follow-up of simple febrile convulsions. EEG is only important in elucidating complicated seizures. Sofijanov et al. series, EEG anomalies were found in 22% of the patients, and it was reported that the frequency of EEG anomalies was higher in complex FS [15]. In the study of Rantala et al., EEG anomaly was detected in 33% of the patients, while no significant difference was found between simple and complex febrile seizures in terms of EEG findings [16]. In our study, an abnormal EEG was observed in four of 44 patients followed up with complex FS, and they were diagnosed with epilepsy. Looking at the literature, some studies suggest that anemia may be a protective factor for febrile convulsions [17], while some studies suggest that there is no relationship between anemia and febrile seizures [18]. Some studies have shown that anemia may be a risk factor for febrile seizures [19-21]. In our study, however, no significant anemia was observed in patients followed up with FS.

## Conclusions

Upper respiratory tract infection, family history of FS, and family history of epilepsy are the main risk factors for the development of FS. Complex FS is a serious risk factor for the development of epilepsy.

## References

[1] (2008). Febrile seizures: clinical practice guideline for the long-term management of the child with simple febrile seizures. Pediatrics.

[2] Nelson KB, Ellenberg JH (1978). Prognosis in children with febrile seizures. Pediatrics.

[3] Nakayama J (2009). Progress in searching for the febrile seizure susceptibility genes. Brain Dev.

[4] Berg AT, Shinnar S, Shapiro ED, Salomon ME, Crain EF, Hauser WA (1995). Risk factors for a first febrile seizure: a matched case-control study. Epilepsia.

[5] Abuekteish F, Daoud AS, al-Sheyyab M, Nou'man M (2000). Demographic characteristics and risk factors of first febrile seizures: a Jordanian experience. Trop Doct.

[6] Syndi Seinfeld D, Pellock JM (2013). Recent research on febrile seizures: a review. J Neurol Neurophysiol.

[7] Okumura A, Uemura N, Suzuki M, Itomi K, Watanabe K (2004). Unconsciousness and delirious behavior in children with febrile seizures. Pediatr Neurol.

[8] Knudsen FU (1996). Febrile seizures-treatment and outcome. Brain Dev.

[9] Heydarian F, Bakhtiari E, Yousefi S, Heidarian M (2018). The first febrile seizure: an updated study for clinical risk factors. Iran J Pediatr.

[10] Abd Ellatif F, El Garawany H (2002). Risk factors of febrile seizures among preschool children in Alexandria. J Egypt Public Health Assoc.

[11] Mahyar A, Ayazi P, Fallahi M, Javadi A (2010). Risk factors of the first febrile seizures in Iranian children. Int J Pediatr.

[12] Annegers JF, Hauser WA, Elveback LR, Kurland LT (1979). The risk of epilepsy following febrile convulsions. Neurology.

[13] Berg AT, Shinnar S (1996). Unprovoked seizures in children with febrile seizures: short-term outcome. Neurology.

[14] Pavlidou E, Panteliadis C (2013). Prognostic factors for subsequent epilepsy in children with febrile seizures. Epilepsia.

[15] Sofijanov N, Emoto S, Kuturec M (1992). Febrile seizures: clinical characteristics and initial EEG. Epilepsia.

[16] Rantala H, Uhari M, Tuokko H (1990). Viral infections and recurrences of febrile convulsions. J Pediatr.

[17] Kobrinsky NL, Yager JY, Cheang MS, Yatscoff RW, Tenenbein M (1995). Does iron deficiency raise the seizure threshold?. J Child Neurol.

[18] Momen AA, Hakim ZM (2003). Case control study of relationship between anemia and febrile convulsion in children between 9 months to 5 years of age. Jundishapur Sci Med J.

[19] Sadeghzadeh M, Khoshnevis Asl P, Mahboubi E (2012). Iron status and febrile seizure- a case control study in children less than 3 years. Iran J Child Neurol.

[20] Heydarian F, Vatankhah H (2012). The role of anemia in first simple febrile seizure in children aged 6 months to 5 years old. Neurosciences.

[21] Papageorgiou V, Vargiami E, Kontopoulos E (2015). Association between iron deficiency and febrile seizures. Eur J Paediatr Neurol.

